# Surgery of the Genito-Urinary Organs

**Published:** 1894-03-03

**Authors:** 


### SURGERY OF THE GENITO URINARY ORGANS.

*Moveable Kidney.*—It is not many years since the importance, if not the very existence, of such a condition as moveable kidney was denied, and now we are told by Dr. Mathieu[1] that in young women between fifteen and twenty years of age 12 or 13 per cent. have moveable kidneys, while the same condition is met with in 22 or 23 per cent. of those between twenty and twenty-five years, and in about 40 per cent. between twenty-five and fifty years, while in older patients the proportion falls again to 25 per cent. He emphasises the importance of a previous pregnancy as a factor in the causation of moveable kidney, and still more the frequency of association of dyspepsia with renal mobility. Instability of the nervous system is a common accompaniment of the affection.

Mr. Hastings Gilford[2] is no less definite in his belief in the frequency of "moveable kidney." He discusses the question of a resultant hydronephrosis, and relates cases where the colon took an abnormal course, which might have had an influence in causing undue mobility of the kidney. Probably the frequency of the occurrence of this condition has been exaggerated, but there is no doubt that it is common, that it gives rise to or is associated with dyspeptic troubles and various ailments of the "neurotic class," and that it is an undoubted cause of hydronephrosis with its resultant evils. Moveable kidney is no longer an imaginary disease; it is a serious ailment, frequently calling for surgical treatment, but it is at present passing through the fashionable stage; it will no doubt find its true level before long.

Is nephrectomy for malignant disease a justifiable operation, and especially is it so in the case of children? These have been debateable questions, and some valuable evidence is now forthcoming on the side of those who are loth to refuse relief to such cases. Dr. Robert Abbe[3] records three cases of nephrectomy for tumour, two in children, one in an adult. In one child of two years the tumour, after removal, was described as an *adeno carcinoma sarcomatosum*; in the other case, a child of fourteen months, there was a *rhabdo-myo-sarcoma* weighing half as much as the child. In both cases a transverse incision was made; in both Trendelenburg's position was maintained during the operation, and is strongly advocated by the author, and in both a good recovery followed. In the third patient, a man of sixty-five, the tumour was a sarcoma, "mostly round" celled, and death followed fourteen hours after operation. In one of Dr. Abbe's successful cases a large part of the kidney was preserved, the growth being mainly extra renal.

While congratulating Dr. Abbe on his successes, it is well to bear in mind that his cases hardly enable us to say that nephrectomy for tumour in children is an operation worth doing, since it appears that the exact nature of the growth in one of his cases is somewhat doubtful, and in the other the tumour appears to have been of the congenital, questionably malignant, and extra renal class; hence the absence of recurrence in these cases is not a conclusive argument in favour of operation as a rule. The contribution is, however, a very important one.

Aldibert[4] discusses the renal surgery of childhood. He believes that in ruptured kidney the peritoneal cavity is often opened and intraperitoneal bleeding occurs, hence he advises transperitoneal operation. He points out that so-called traumatic hydronephrosis is really rather a localised perinephric collection of urine. For congenital unilateral hydronephrosis primary nephrectomy is shown to be more successful than other modes of treatment. Dr. Howard Kelly[5] relates a successful case of "uretero-ureterostomy," or more intelligibly one in which, after an unintentional division of the ureter in an operation for uterine myoma, he united the two ends of the duct by invagination of the upper end into the lower through a slit made in the latter after closure of its upper aperture. The mode of producing invagination and securing the union of the two ends resembles somewhat Paul's operation for uniting divided segments of intestine.

It appears that, in spite of the obvious objections to such an operation, catheterism of the male ureters is still attempted from time to time, and Dr. J. Brown,[6] of Baltimore, records his observations upon the proceeding, which is happily admitted to be applicable only to certain cases.

*Hydronephrosis.*—The importance of hydronephrosis is, in Dr. D. W. Graham's[7] opinion, very great, since not only may there be mental disquietude, but also "functional disturbance of every neighbouring organ." He considers it to be a "progressively destructive" condition, and believes that as it is a frequent sequel to mobility of the kidney, that condition should be dealt with by nephrorraphy even when no very severe symptoms are present.

A case of acute intermittent hydronephrosis from valvular stricture of the ureter is described by Dr. H. Mynter[8], who remedied the defect by a plastic operation.

An interesting account of experimental and clinical observations on the production of hydronephrosis by "kinking" of the ureter of a moveable or misplaced kidney is given by Dr. Tuffier[9], who strongly advocates nephropexy in such cases.

In a discussion on hydronephrosis, Dr. Bazy[10] mentioned that he had shown experimentally that complete obliteration of the ureter might give rise to hydronephrosis instead of to arrest of renal secretion. This now well-established fact is of great importance in renal surgery.

An important paper on undue frequency of micturition in women is contributed by Dr. Herman,[11] who gives the following causes for the affection—(1) Certain nervous diseases, *e.g.*, ataxy, in which the bladder is hyperæsthetic; (2) a congenitally small bladder; (3) over-distention of the bladder on some one occasion;

(4) excessive quantity of urine; (5) the group of cases subject to nocturnal incontinence; (6) retention of urine; (7) irritation of the bladder by a pessary, by superficial ulceration of the urethra, &c., or by more remote conditions, *e.g.*, piles; (8) weakness or prolapse of the pelvic floor or contents resulting from child-bearing; (9) "dislocation of the urethra" by pressure or traction; (10) stricture of the urethra or congenitally small meatus; (11) cystitis or kidney disease.

In nocturnal incontinance of children Freud[12] has noticed a spastic condition of the adductors and quadriceps of the thigh as an associate condition.

The radical treatment of tumours of the bladder is advocated by Bazy,[13] who considers that all such growths are removable, and in many cases should be removed, or, failing this, the bladder should be opened and drained. After removal of the tumour by excision, the wound should be, if possible, sutured.

*Vesical Calculus.*—R. J. Pye Smith[14] relates his personal experience of the method of dealing with very large vesical calculi, by breaking them up by the hammer and chisel, or other analogous means, after making a lithotomy incision.

An addition to the list of cases of recovery after suture of an intraperitoneal rupture of the bladder is made by Dr. Thomas Kerr,[15] who appends a list of the recorded cases.

In a clinical lecture on suprapubic cystotomy, F. A. Southam[16] illustrates some of the conditions which call for this operation, such as the presence of several hard calculi in a patient suffering from enlarged prostate, who was also suspected of renal disease. In another case there was, in a boy of eleven, a fair-sized hard calculus. The suprapubic operation was preferred to lithotrity on account of the boy's intolerance of any manipulations. Difficulty in introduction of the instruments led in another instance to the selection of the high operation in preference to lithotrity. The removal of a large growth in a woman was successfully accomplished by the suprapubic route, and finally an interesting series is closed by mention of a case of chronic cystitis, in which the suprapubic opening allowed the presence of a pouch with surrounding ulceration to be discovered.

The question of the employment of litholapaxy in boys is discussed at length by Surgeon-Major Dennys,[17] who adds to the experience of the operation, and supports the observations of Keegan, Freyer, and others as to the general applicability of the operation, which, it is now well recognised, holds an important place in the surgery of childhood.

[1] Medical Week, Dec. 15, 1893. [2] Lancet, Dec. 23, 1893. [3] Annals of Surgery, Jan., 1894. [4] Rev. Mans. des Maladies de l'Enfance tome xi., 1893. [5] Annals of Surgery, Jan., 1894. [6] Annals of Surgery, Jan., 1894. [7] Practitioner, Dec., 1893. [8] Annals of Surgery, Dec., 1893. [9] Med. Week, Dec. 15, 1893. [10] Med. Week, Dec. 1, 1893. [11] C.in. Journ., Dec. 27, 1893. [12] Brit. Med. Journ., Dec. 9, 1893. [13] Rev. de Therap. Medic. Chirurgicale, Nov. 5, 1893. [14] Quarterly Med. Journ., Oct., 1893. [15] Aunals of Surgery, Dec., 1893. [16] Brit. Med. Journ., Jan, 13, 1894. [17] Lancet, Dec. 9, 1893.

